# Investigating the Pregnancy and Post-Partum Health Experiences of Women Living with HIV

**DOI:** 10.1007/s10995-024-03962-y

**Published:** 2024-06-21

**Authors:** Rebecca Cooper, Julia Greig, Hilary Piercy, Paul Collini

**Affiliations:** 1 The University of Sheffield, Sheffield, England; 2 Department of Infectious Diseases and Tropical Medicine Sheffield Teaching Hospitals, The University of Sheffield, Sheffield, England; 3 Sheffield Hallam University, Sheffield, England

**Keywords:** HIV, Infant feeding, Social support, Neonatal screening, Pregnancy, Post-partum, Breastfeeding

## Abstract

**Introduction:**

Pregnancy and the postpartum period is a difficult time for women living with HIV (WLWH) and postpartum engagement with HIV care is often reduced, with implications for health and well-being. We aimed to explore the postpartum health experiences of WLWH in relation to engagement in HIV care.

**Methods:**

The NESTOR (iNvESTigating the pregnancy and pOst-paRtum health experience of women living with HIV) study was a UK based qualitative semi-structured interview study. 61 eligible women were identified. We used a purposive sampling technique to recruit women with differing levels of engagement in HIV care. Interviews were conducted via telephone or video call. Interviews were audio recorded and fully transcribed. We used a thematic approach for data analysis, and two researchers independently coded the data and established the key themes.

**Results:**

11 of 61 (18%) eligible women participated in the interviews, and the three main themes were ‘infant feeding decisions’, ‘managing the risk of mother to child transmission’, and ‘managing the knowledge of their HIV status’. These themes offer detailed insights into the significant psychological and emotional challenges these women had experienced, and the practical support from healthcare professionals in both HIV and maternity services that had enabled them to navigate those challenges.

**Discussion:**

There have been life-changing developments in the treatment and care for people living with HIV. However, even in the U = U (undetectable = untransmittable) era, traditional concerns about breastfeeding, risk of transmission to the infant and stigma continue to shape the postpartum experience of WLWH. As these impact on their emotional and psychological wellbeing, support in these areas needs to be prioritised.

## Significance

Studies report that postpartum retention in HIV care and viral load suppression are suboptimal for women living with HIV, where an important reason is the financial cost and access to healthcare. However, there is a relative scarcity of evidence describing the problems faced by women accessing HIV and maternity services within socialised healthcare systems, such as those in the UK.

Women living with HIV face unanticipated challenges when becoming new mothers relating to disclosure of their HIV status, fears of vertical transmission and perceived perceptions of others deeming them inadequate mothers. Importantly, our analysis illustrates potential opportunities where intervention might mitigate the emotional and physical impact of these issues on their wellbeing throughout pregnancy and postpartum.

## Introduction

In 2020 approximately 38.4 million people worldwide were living with HIV, including 1.7 million children (UNAIDS, 2021). The establishment of antiretroviral therapy (ART) has dramatically improved life expectancy and quality of life and provides a major contribution to reduction in new infections, especially mother to child transmission (Harris & Yudin, 2020). In 2021, 81% of women living with HIV (WLWH) had access to ART during pregnancy and childbirth (UNAIDS, 2021). Peripartum transmission rates are reduced to < 1% when a pregnant woman is on ART with an undetectable HIV viral load (< 40 copies/mL) (Kourtis et al., 2006; Montgomery, 2003). The PROMISE trial showed that continuing ART in the postpartum period further reduces the risk of transmission whilst breastfeeding to 0.3% at six months and 0.6% at 12 months (Durban & Africa, 2018).

Current United Kingdom (UK) guidelines recommend that all infants born to WLWH should not be breastfed to minimise the transmission risk (BHIVA, 2020). It is identified that abstaining from breastfeeding can come at an emotional, financial, and social cost, so multidisciplinary team (MDT) support should be provided (BHIVA, 2020). Nonetheless, if WLWH still choose to breastfeed accepting the small risk of transmission, they should be supported to do so, providing they meet certain criteria (Sect. 9.4.4 of the UK guidelines) and agree to increased clinical reviews (BHIVA, 2020).

Nine of 15 studies in a systematic review revealed concerns regarding transmission risk remain, and that it was extremely important to women that this was reduced (Lytvyn et al., 2017). Key to this, is engaging with HIV treatment and care during pregnancy and postpartum, however studies from the United States (US), Brazil and the UK report that postpartum retention in HIV care (e.g. only 39%, (Adams et al., 2015) and viral load suppression (e.g. adjusted hazard ratio for viral rebound 2.63 (1.58–4.39) (Huntington et al., 2015) are significantly lower for WLWH compared to non-pregnant controls (Adams et al., 2015; Hoffmann et al., 2016; Huntington et al., 2015; Loftus et al., 2016). Studies from WLWH in Sub-Saharan Africa (SSA) have found that higher clinic retention rates relate to the desire to maintain their own health and prevent transmission (Knettel et al., 2018). A study of a peer support intervention in London conducted qualitative interviews to describe experiences of mothers living with HIV, and found three key themes of ‘stigma and isolation’, ‘fear and distress’ and the ‘gap in maternity care’ (McLeish & Redshaw, 2016).

Thus, physical and emotional factors make pregnancy and postpartum a turbulent time for WLWH and impacts on their wellbeing. However, the evidence in this field is largely derived from African and US studies, with a relative scarcity of evidence originating from Europe, where HIV and maternity services are within socialised healthcare systems. The NESTOR study (iNvESTigating the pregnancy and pOst-paRtum health experiences of women living with HIV) aimed to obtain first-hand in-depth insight into the health experiences of WLWH when having a baby to investigate further whether these findings are generalisable, and applicable to the UK setting.

## Methods

The NESTOR study was a descriptive, qualitative study using semi-structured individual interviews to explore the health experiences of postpartum WLWH.

We invited any adult woman currently registered with an NHS HIV clinic in South Yorkshire, UK, who had given birth while registered in the same service in the period 1st January 2012–31st December 2019, and were at least 12 months postpartum, to take part until we had enrolled a minimum of 10 participants. As these women, all English speakers, had previously been identified in an internal audit of clinic attendance and viral load suppression we used purposive sampling aiming to include WLWH across the spectrum of engagement with care. Their HIV nurse contacted them by telephone to explain the purpose of the study and invite them to participate. A patient information sheet and informed consent form were then sent by post, followed up with a telephone call to arrange the interview.

Interviews took place between March 2021 and August 2021. All were conducted in English by a single member of the research team (RC) via telephone or video call and using a topic guide to structure the interview. Consent was received at the start of the call and documented by the researcher, interviews proceeded in the presence of a clinical staff member. There was no compensation for travel or time contributions. Interviews lasted from 25 to 75 min. RC fully transcribed and anonymised the interviews. We also extracted the following from the clinical records: ethnicity, country of birth, age at delivery, viral load postpartum, appointments attendance and reported psychosocial problems (pertaining to mental, social, emotional, or spiritual aspects of their life) in the 12-months postpartum. Detectable viral load was defined as a HIV RNA > 50 copies/ml (or equivalent).

Two researchers (RC and HP) independently analysed the interview transcripts using thematic analysis (Braun & Clarke, 2006). Both researchers independently coded the data sets using NVivo digital software (QSR International, 2020), further refining codes as more specific themes began to emerge. They then performed a detailed, collaborative thematic analysis using an iterative process to determine the most encompassing, comprehensive themes.

### Ethical Approvals

The NESTOR study was approved by South Yorkshire research ethics committee’s (REC 20/YH/0329) and the UK Health Research Authority (HRA).

## Results

Of the 61 identified patients, we invited 36 WLWH to enrol in the study. Five had a detectable viral load during pregnancy or postpartum, one of whom (20%) consented to enrol. 47% (17) of invited women had a documented psychosocial problem and 47% had a history of missed appointments, 35% (6/17) and 24% (4/17) respectively of whom consented to enrol. In total 11 WLWH were enrolled. Main reasons for declining enrolment were being unable to commit time and not wanting to discuss personal health. Median age at delivery was 40 (32–50) years. Nine women were of SSA origin, one European and one from the UK. All were taking antiretrovirals when they conceived, and all engaged with HIV services during their pregnancy. They had a total of 14 pregnancies, as three women gave birth twice in the seven year period. Two had a detectable viral load and one had no viral load data in the first 12 months postpartum. All pregnancies resulted in single live births with no vertical transmission of HIV. All the women had been diagnosed with HIV prior to their pregnancy. No women decided to breastfeed.

We identified three key themes, each with two sub-themes.

### Theme 1 Infant Feeding Decisions

Every woman struggled with the knowledge and the decision that they would not be able to breastfeed, often despite a strong personal desire to breastfeed. They were also acutely aware of the stigma surrounding not breastfeeding in their community. As participant (P)10 said, “I would have loved to be able to feed my child with my body, but it just wasn’t an option, so I just had to get my head round that”.

#### Subtheme: Making the Decision

The prospect of bottle feeding was difficult for many, particularly those from SSA who saw breastfeeding as a cultural norm

“That is very, very difficult. Especially in the African people, because we don’t believe mainly in bottle feeding, and especially if you are not going to work” (P1, SSA).

Several identified the feelings of guilt associated with their decision to bottle feed

​​“…you feel that by not breastfeeding you’re not caring as much for your child or giving him a good start in life.” (P8, SSA).

“…there was some guilt because you have this milk, and you can’t use it for your baby” (P6, SSA).

The degree to which women were reconciled to their decision varied. Some had had few difficulties accepting the advice

“I understood that it’s best for the baby… to not breastfeed, so I had no problem with that” (P9, SSA).

Others were much less comfortable with the situation, but felt they had no option but to ignore their own wishes and cultural norms

“[staff at the maternity hospital said] ‘We haven’t had anybody who has been insisting on breastfeeding, so we are not comfortable if you breastfeed’… so, in the end I didn’t [breastfeed] because that’s what [the medical staff] wanted, not what I wanted” (P5, SSA).

#### Subtheme: Managing the Social Pressure

Explaining their infant feeding decision to their friends and family without disclosing their HIV status came with great difficulty. Many women expressed concerns that they had to lie to do this:

“I lie, because I do not want to explain my problem.” (P3, SSA)

“I feel guilty because I’m lying about why I don’t breastfeed” (P8, SSA)

Some women’s decision to not breastfeed were challenged by others:

“I’ve had people say to me, I’m such a bad mother, I’ve had people say that I am trying to keep my shape that’s why I am not breastfeeding” (P2, SSA)

A common strategy to manage this social pressure was the creation of a cover story, to hide why they could not breastfeed:

“I’ve also felt the need to come up with a story… I don’t feel that I can speak to other people about it, I don’t think people really understand without judging” (P8, SSA)

One participant stated that it would have been beneficial to have a healthcare professional take time to ensure they were prepared to field questions about why they were not breastfeeding:

“It would have been good to talk about it in case somebody had asked [why I didn’t breastfeed]… and for your own piece of mind, having your story straight.” (P11, Europe).

### Theme 2 Managing the Risk of Mother-to-Child Transmission

This theme explored the risks of transmission associated with HIV and pregnancy and how mothers and the healthcare professionals responsible for their care sought to reduce them.

#### Subtheme: Concerns of Transmission

The transmission risk remained poorly understood by many women, creating further anxieties and remaining the greatest concern for all of the women.

“I didn’t know that there was medicine as well for kids and I didn’t know that when I am taking the tablet, [my viral load can be undetectable] so that it is not affecting the child” (P4, SSA)

“Just things like if I had a cut, if I’m sneezing on the baby…or even sometimes if you’re cuddling the baby and if I would sweat, I think it was more paranoia” (P6, SSA)

Women valued the support of medical staff who alleviated their anxieties and fears relating to transmission.

“I was just concerned…but after talking to the doctors, they told me it won’t be transmitted, [because of ARVs]. So, I was happy because I didn’t want my child, my kids to have the condition.” (P4, SSA)

“…the clinic has always been supportive in sort of saying [having a child] will be fine, which is what I needed to hear.” (P11, Europe)

The emotional impact of these worries persisted for many throughout their pregnancy, and often did not ease until they received final confirmation that their child did not have HIV:

“I was kind of nervous about the pregnancy, then I’ve got [the neonatal testing] on top of it so until I got that final 18-month tick in the box it was quite a stressful experience.” (P10, UK)

#### Subtheme: Managing the Risk of Transmission

Administering post-exposure prophylaxis to the neonate caused profound anxieties

“…there was a time when I missed a dose, but I panicked. I felt like I had literally dropped her on the floor.” (P7, SSA)

“…when the four weeks were over, I just thought, ‘thank God’, I don’t have the pressure anymore, watching the clock, setting alarms to give her medication, so I was so happy that I’d ticked all the boxes.” (P7, SSA)

Despite this pressure, the women expressed gratitude towards the staff who alleviated these difficulties

“I think there was a little bit of anxiety on my part about administering the medication… but [the medical staff] were all really, really good and they coached me all the way through it.” (P10, UK)

A lack of communication was reported by one, who described the fear of disclosure and/or being asked unwanted questions whilst attending appointments

“I thought we were just going in and out of the hospital getting bloods, but it was all the way in and again there was some anxiety about bumping into people who also had babies at the same time… so my worry I suppose was what if I see somebody” (P11, Europe)

### Theme 3 Managing the Knowledge of their HIV Status

An underlying theme running through all interviews was the stigma relating to HIV and fear of disclosure of their HIV status, revealing the depth of the social struggles and emotional impact of the stigma surrounding HIV.

#### Subtheme: Threats to Disclosure

The majority of the women involved with this study had disclosed their HIV status to only a few people, so suddenly having new members of the medical team aware of their HIV diagnosis became very daunting:

“…it suddenly dawned on me that I’m now pregnant, I’m supposed to be going to [maternity hospital], I’m going to be meeting all these people, you know I kind of panic, because there’s still a bit of stigma around HIV”. (P7, SSA)

“Everybody is different, but I have always been very private about my health and very few people know about my HIV, so it was really difficult to… feel that there were more people in the circle of trust because I was pregnant… and then nurses and everybody knowing, so I felt like, almost like I couldn’t quite hold my information”. (P11, Europe)

There were mixed opinions on whether support groups such as ‘mum and baby’ groups for women with HIV should exist

“Going to a group would mean someone else knows… I would have to share my status with somebody who I don’t know in that situation would keep my private information”. (P11, Europe)

“At some point I could have done with meeting other mothers who are HIV positive and have babies… It would have been nice to go to a play group, or mother and child group with women that are HIV who have had babies and stuff like that” (P9, SSA)

#### Subtheme: Minimising the Risk of Disclosure

The anxieties of people learning of their HIV status was heightened during the postpartum period; women described several situations they believed posed a disclosure risk

“what I went through to hide the labels, I worked with my sister, we got home and we scrubbed off the labels [of the medication] with a wire brush, we literally tore off everything that was HIV related”. (P7, SSA)

“I was introduced to a [peer support] group, which I feel like is kind of exposing me, you know”. (P2, SSA)

The women were grateful for attempts to help maintain the confidentiality of their HIV status. The provision of a private room was often appreciated:

“they were able to give me my own room, so I was okay to talk about [my HIV openly] when people came in”. (P10, UK)

“Or having to worry if somebody else talked about it, you know, thinking well I didn’t want you to say that, maybe, and having a private room felt like it was, it was a safe space, you know”. (P11, Europe)

In general, reports from the women regarding the services for HIV and antenatal care were very positive, and described subtle ways the help and attitudes made them more confident engaging with their HIV care:

“I used to feel really anxious when I went in to the [clinic]… but… everybody is so positive about you know even your diagnosis, your status, your having babies, having children, having a normal life, that you always leave feeling good, and positive and like most of the stigma is taken away”. (P10, UK)

“… for me, going to the clinic is always really hard and I want to be in and out quick and I knew that the people knew that and they would kind of do as best as they can to make it an okay experience”. (P11, Europe)

## Discussion

In our study the chief concerns during pregnancy and post-partum for WLWH clustered into three main themes, regarding infant feeding decisions, managing the risk of mother-to-child transmission and managing the knowledge of their HIV status.

The importance of, and difficulties regarding infant feeding decisions, and their pre-eminence among those from SSA cultures, has received little attention in high-income settings. Our participants described difficulties explaining their decisions to not breastfeed, and the ensuing social pressure from family and friends. This pressure derived from a pernicious combination of the visible - stigma from contravening cultural norms to breastfeed, and the invisible - stigma of HIV status disclosure, as previously described in WLWH in high-income settings (Odeniyi et al., 2020). Whilst all women described these difficulties, we noted that cultural norms of breastfeeding made the decision to not breastfeed much more significant for the African women, who described bottle feeding children as infrequent in their communities. Participants also described many positive interventions and attitudes from medical staff to make them feel more comfortable, including providing a private room after delivery (as advised by UK guidelines)(BHIVA, 2020), which facilitated speaking openly about their medical condition and concealing the fact that they hadn’t attempted to breastfeed. The benefits of breastfeeding are well recognised as are the reasons women may still choose to breastfeed against medical advice, particularly among those from sub-Saharan Africa where it is the cultural norm (Moseholm & Weis, 2020). Apparently conflicting guidelines and messaging also caused confusion; in low-income settings, where many of our WLWH originated from, breastfeeding is recommended and encouraged while women, aware of the 2016 Undetectable = Untransmittable (U = U) campaign, question why this doesn’t relate to breastfeeding (Moseholm & Weis, 2020). The heavy focus on bio-medical issues relating to breastfeeding and transmission has overshadowed the social and cultural difficulties that WLWH who can’t breastfeed face (Waitt et al., 2018). A 2018 UK paper following the U = U breakthrough argued for a greater evidence base before recommending to women that they should not breastfeed (Waitt et al., 2018). A recent US survey of HIV health care providers found that 75% had been asked by a WLWH if she could breastfeed, and 29% had cared for a patient who chose to breastfeed against recommendation (Tuthill et al., 2019). The number of women choosing to breastfeed in high-income countries is likely to increase over time, yet there is still a lack of data on HIV transmission through breast milk in high-income countries (Moseholm & Weis, 2020; Tuthill et al., 2019). 100% of the women in the NESTOR study expressed that they would have breastfed if they didn’t have HIV, yet none chose to do so. Adding an infant feeding section to birth plans could act as a prompt for clinicians to initiate and document the infant feeding conversation and provide opportunity for WLWH to ask transmission specific questions and make an informed decision about breastfeeding vs. bottle feeding. Encouraging the use of patient information leaflets can also allow women to make this decision in their own time without perceived pressure from outside influences. Such leaflets already exist in the UK (BHIVA, 2020).

Managing the knowledge of their HIV status pervaded every aspect of participants’ care. It was variously expressed as fear of staff accidentally disclosing, friends and family discovering HIV was the true reason for not breastfeeding and an apprehension of the social impact from people knowing their status. HIV stigma and status disclosure feature prominently in studies of WLWH, having negative impacts not only socially, but also on health and wellbeing (Rizza et al., 2012). For example, pregnant and postpartum WLWH may be reluctant to attend clinics for fear of being seen and revealing their HIV status (Hodgson et al., 2014). Our WLWH described not wanting to enter clinics through certain entrances nor wanting to wait too long. SSA WLWH in our study expressed beliefs and worries that disclosure of their HIV status would risk them being perceived as incapable mothers and undermine their roles as homemakers, in keeping with existing literature (Hodgson et al., 2014). Although our study could not directly measure it, such fear of disclosure can lead to more missed appointments and reduced adherence to medication (Knettel et al., 2018). Correspondingly, perceived negative attitudes from health workers have been identified as a barrier to ART adherence for pregnant and postpartum WLWH, whereas non-judgemental approaches from healthcare staff have a positive impact on adherence (Hodgson et al., 2014; Phillips et al., 2014). Notably, although comments about healthcare staff in general were extremely positive and gracious, some new mothers felt that maternity unit staff neither understood their HIV nor appeared comfortable around them. Thus, added to the concern of transmission during pregnancy and postpartum is the turmoil from fear of disclosure and stigma presented by the new, yet necessary, interactions with perinatal health services. This was equally worrisome for women of sub-Saharan African and non-African heritage. Reflecting the literature, many women voiced that their urge to not transmit HIV to their child provided extra motivation to engage, adhere to ART and maintain viral suppression (Hodgson et al., 2014; Lytvyn et al., 2017; Raffe et al., 2017). Ensuring healthcare staff are offered up-to-date HIV and stigma training is crucial to improving the health experiences of WLWH. New training programmes are being developed and delivered in the UK to begin tackling these negative experiences (NHS England, 2023).

The most significant limitation of this study was the lack of women with poor engagement in care and adherence. A previous audit in this service identified pregnancy and post-partum WLWH with ‘poor engagement’, but despite every effort, most were not recruited, thus limiting our scope to gather information from women that may have faced significant challenges. Furthermore, while we recruited participants representative of the age and ethnicity of WLWH in the UK, as a single centre with a relatively small sample size we cannot assume our observations have encompassed all relevant factors. We suggest national scale surveys in the UK and beyond, informed by the present study and that include the broadest demographic, are now indicated. By confirming, and expanding on, the generalisability of our findings these will catalyse the introduction of improved approaches that address the needs of WLWH during pregnancy and post-partum.

## Conclusion

WLWH must cope with many social, emotional, and financial consequences in the postpartum period. Concerns of disclosure and HIV stigma reflect those described in all PLWH but with additional intensity related to the new and increased interactions with health care services from becoming new mothers. Improved understanding of the concerns of WLWH during this period can help shape further support. Healthcare staff need to be more open and clearer about the balance of evidence on HIV transmission through breastfeeding during the infant feeding decision conversations and should look to support more women to breastfeed where this is truly important to them. Whilst anxiety about risk of transmission can never be entirely removed, reassurance and support appear to be a significant factor contributing to improved emotional and physical wellbeing of WLWH. Service improvement should provide HIV-specific training to staff in maternity care including stigma awareness and communication with WLWH.
